# CD9^+^ Regulatory B Cells Induce T Cell Apoptosis via IL-10 and Are Reduced in Severe Asthmatic Patients

**DOI:** 10.3389/fimmu.2018.03034

**Published:** 2018-12-21

**Authors:** Carole Brosseau, Maxim Durand, Luc Colas, Eugénie Durand, Aurore Foureau, Marie-Aude Cheminant, Gregory Bouchaud, Laure Castan, Martin Klein, Antoine Magnan, Sophie Brouard

**Affiliations:** ^1^Centre de Recherche en Transplantation et Immunologie UMR 1064, INSERM, Université de Nantes, Nantes, France; ^2^Institut de Transplantation Urologie Néphrologie, CHU Nantes, Nantes, France; ^3^Institut du thorax, Inserm UMR 1087, CNRS UMR 6291, Université de Nantes, Nantes, France; ^4^Institut du Thorax, CHU de Nantes, Nantes, France; ^5^Faculté de Médecine, Université de Nantes, Nantes, France; ^6^INRA Centre Angers-Nantes, Nantes, France; ^7^Centre d'Investigation Clinique (CIC) Biothérapie, CHU Nantes, Nantes, France

**Keywords:** regulatory B cells, CD9^+^ B cells, severe asthma, apoptosis, effector T cells

## Abstract

CD9 was recently identified as a marker of murine IL-10-competent regulatory B cells. Functional impairments or defects in CD9^+^ IL-10-secreting regulatory B cells are associated with enhanced asthma-like inflammation and airway hyperresponsiveness. In mouse models, all asthma-related features can be abrogated by CD9^+^ B cell adoptive transfer. We aimed herein to decipher the profiles, features, and molecular mechanisms of the regulatory properties of CD9^+^ B cells in human and mouse. The profile of CD9^+^ B cells was analyzed using blood from severe asthmatic patients and normal and asthmatic mice by flow cytometry. The regulatory effects of mouse CD9^+^ B cells on effector T cell death, cell cycle arrest, apoptosis, and mitochondrial depolarization were determined using yellow dye, propidium iodide, Annexin V, and JC-1 staining. MAPK phosphorylation was analyzed by western blotting. Patients with severe asthma and asthmatic mice both harbored less CD19^+^CD9^+^ B cells, although these cells displayed no defect in their capacity to induce T cell apoptosis. Molecular mechanisms of regulation of CD9^+^ B cells characterized in mouse showed that they induced effector T cell cycle arrest in sub G0/G1, leading to apoptosis in an IL-10-dependent manner. This process occurred through MAPK phosphorylation and activation of both the intrinsic and extrinsic pathways. This study characterizes the molecular mechanisms underlying the regulation of CD9^+^ B cells to induce effector T cell apoptosis in mice and humans via IL-10 secretion. Defects in CD9^+^ B cells in blood from patients with severe asthma reveal new insights into the lack of regulation of inflammation in these patients.

## Introduction

Beyond their capacity to secrete antibodies, B cells are also able to produce cytokines (1), express major histocompatibility complex and co-stimulatory molecules (2), present antigens (3), and regulate T-cell-mediated immune responses (4). Depending on the specific subset, B cells may thus promote, enhance, and/or regulate inflammation depending on the pro- and anti-inflammatory cytokines secreted, the balance of which influences the immune response (4). It is now well-established that specific B cell subtypes are involved in the maintenance of homeostasis of the immune system and regulate inflammation in pathological situations (5, 6). Also called regulatory B cells (Bregs), these specific B cells have immunosuppressive properties; their functional impairment is associated with exacerbated and/or persistent autoimmune processes (7–11), whereas their presence correlates with a state of tolerance in transplantation (12–17).

Although the presence and role of Bregs are thus clearly evidenced in different models and pathologies, their full molecular characterization remains elusive, primarily because no specific markers or transcription factors have been identified in rodents and humans (18). To date, the most commonly used marker is IL-10 secretion (7, 18). Interestingly, allergic patients display a lower frequency of IL-10-secreting Bregs with altered function than healthy volunteers (HV) or allergen-tolerant patients (19, 20). Patients with allergic asthma harbor a defective expansion of such IL-10–producing B cells in response to lipopolysaccharide (LPS) stimulation and a weaker IL-10 response to house dust mite (HDM) allergen–activated T cells (21–23). We have previously demonstrated that murine IL-10^+^ Bregs are enriched in a CD9^+^ B cell subset (24). Induction of allergic asthma in mice alters the homeostasis of IL-10^+^ Bregs, and adoptive transfer of CD9^+^ B cells alone is sufficient to abrogate asthma in an IL-10-dependent manner (24). Finally, we have shown that CD9 expression in humans is dramatically increased at the surface of CD24^hi^CD38^hi^ immature B cells, thus defining an important IL-10 Breg subset (24). This finding has been confirmed by others (25–27), and CD9 thus appears to be a reliable marker for defining both mouse and human Bregs.

We show herein, for the first time, that mouse and human CD9^+^ B cells elicit regulatory properties through IL-10 secretion, and transitional CD24^hi^CD38^hi^B cells expressing CD9 are decreased in the blood of severe asthmatic patients. We report that CD9^+^ B cells induce effector T cell apoptosis via the secretion of IL-10. In mouse, T cell proliferation is blocked at the sub G0/G1 cell cycle phase, leading to activation of the intrinsic and extrinsic apoptotic pathways via a MAPK-dependent mechanism. These data reveal new insights on the lack of regulation of inflammation in severe asthmatic patients and help pave the way to the discovery of potential novel therapies.

## Materials and Methods

### Asthmatic Patients

This study was performed in accordance with the recommendations of the University Hospital Ethical Committee of Nantes and the Committee for the Protection of Patients from Biologic Risks with written informed consent from all subjects. All subjects provided written informed consent in accordance with the Declaration of Helsinki. The protocol was approved by the University Hospital Ethical Committee of Nantes and the Committee for the Protection of Patients from Biologic Risks. Blood samples were collected from patients included in the EXPRESA clinical study (NCT00721097), which is a prospective cohort of severe asthmatic patients (28). Pulmonary function tests, clinical data, and blood samples were collected each month for 1 year. Nine severe asthmatic patients were selected within EXPRESA cohort based absence of asthma exacerbation in the previous month, absence of systemic corticosteroids (short and long course) and frozen PBMC samples availability. Ten age- and sex-matched Healthy Volunteers (HV), who were free from atopy, asthma (whatever phenotype), allergic rhinitis, atopic dermatitis any other inflammatory diseases, and drugs (whatever route) were used as controls. All severe asthmatic patients had high doses of inhaled corticosteroids (>1,200 microg of beclometasone or equivalent). In HV, % of predicted FEV1 and its coefficient of variation was assumed to 100% (70–130) and age-matched according to reference lung values consensus (29, 30). ACQ7 score was assumed to be at 0 in HV (Supplementary Data Table 1).

### Immunophenotyping of Asthmatic Patient Samples

Peripheral blood mononuclear cells (PBMCs) were isolated by Ficoll-Paque (GE Healthcare, Marolles-en-Hurepoix, France) gradient centrifugation and frozen. Immunophenotyping of PBMCs from 9 severe asthmatic patients was performed using flow cytometry. PBMCs from 10 HV were analyzed as controls. PBMCs were rapidly thawed by placing cryovials at 37°C, washed and stained according to standard protocols using the following mAbs: CD19-BUV395, CD27-BUV737, CD38-BV605, CD24-PerCP-Cy5.5, and CD9-BV510 (BD Biosciences, Le Pont de Claix, France). These markers were used to distinguish CD19^+^ B lymphocytes, CD19^+^CD27^+^ memory cells, CD19^+^CD27^−^ naïve cells, CD19^+^CD24^hi^CD38^hi^ transitional cells, CD19^+^CD24^−^CD38^+^ plasma cells, and CD19^+^CD9^+^ Bregs. For all experiments, dead cells were excluded using the Zombie NIR™ Fixable Viability kit (BioLegend, London, UK). Human anti–IL-10 (BD Biosciences) was used to inhibit the IL-10 pathway. Samples were assessed on a BD LSRFORTESSA X-20 (BD Biosciences, Le Pont de Claix, France), and the data were analyzed using FlowJo v10 software (FlowJo LLC, Ashland, OR, USA).

### Asthmatic Mouse Model

Six week-old wild-type BALB/c mice were purchased from Charles River Laboratories (Ecully, France). Allergic inflammation was induced using a total House Dust Mite (HDM) extract (Dermatophagoïdes farinae) provided by Stallergenes (Antony, France), as previously described (31). This study was performed in accordance with the recommendations of the Regional Ethical Committee for Animal Experiments of Pays de la Loire (ceea.2012.77) under accreditation number 3455. The protocol was approved by the Regional Ethical Committee for Animal Experiments of Pays de la Loire.

### Cell Sorting

Six week-old wild-type BALB/c mice were purchased from Charles River Laboratories (Ecully, France). Splenic cells were isolated and stained with the following antibodies for cell sorting by flow cytometry: CD19-APC-H7 (1D3), CD9-BV421 (KMC8), CD4-FITC (GK1.5) (BD Biosciences, Le Pont-de-Claix, France); and CD3-APC (145-2C11), and CD25-PE (PC61.5) (eBioscience, Paris, France). These markers were used to distinguish CD19^+^CD9^−^ non-regulatory B cells, CD19^+^CD9^+^ Breg cells and CD3^+^CD4^+^CD25^−^ effector T cells. Cells were sorted on a BD FACSARIA III (BD Biosciences, Le Pont-de-Claix, France).

### Cell Activation, Co-culture, and Treatments

Mouse cells (1 million/mL) were cultured for 48 h in RPMI-1640 medium with 10% fetal calf serum and 2 mM glutamine. CD19^+^CD9^−^ non-regulatory B cells or CD19^+^CD9^+^ Breg cells were activated with 2 μg/mL anti-CD40 (HM40-3) (BD Biosciences, Le Pont-de-Claix, France) for 48 h and 10 μg/mL LPS for 5 h. CD3^+^CD4^+^CD25^−^ effector T cells were activated with 100 U/mL interleukin-2 (IL-2) for 48 h. Effector T cells were then co-cultured for 48 h with non-regulatory or Breg cells at a ratio of 1:1 and at a concentration of 1 million/ml on plates coated with anti-CD3 (145-2C11) (BD Biosciences, Le Pont-de-Claix, France). T cells alone were cultured as controls. For human co-culture experiments, the same protocol was used except that B cells were activated with 50 ng/mL recombinant human soluble CD40L (R&D Systems Europe, Lille, France) plus 2.5 mg/mL CpG oligodeoxynucleotide 2006 (InvivoGen, San Diego, CA, USA), and T cells were activated with 50 U/mL recombinant IL-2 (SARL Pharmaxie, Aigueperse, France). During co-culture, the cells were treated with 50 nM Z-VAD (R&D Systems Europe, Lille, France) to inhibit apoptosis. To inhibit IL-10 and the transforming growth factor-beta (TGF-β) and Fas pathways, the cells were treated during co-culture with 10 μg/mL anti–IL-10 (BD Biosciences, Le Pont-de-Claix, France), 10 μg/mL anti–TGF-β1 (Abcam, Inc., Cambridge, UK), or 20 μg/mL anti-Fas ligand (R&D Systems Europe, Lille, France), respectively. To block CD9 function, the cells were treated during co-culture with 0.2 μg/μL KMC8 monoclonal antibody (BD Biosciences, Le Pont-de-Claix, France). To activate the IL-10 pathway, the cells were treated with 10 ng/mL recombinant IL-10 (R&D Systems Europe, Lille, France).

### Viability, Apoptosis, and Cell Cycle Assays

To determine the percentage of dead CD3^+^CD4^+^CD25^−^ effector T cells, the cells were stained using a LIVE/DEAD Fixable Yellow Dead Cell Stain Kit according to the manufacturer's recommendation (Invitrogen, Waltham, US). B cells were removed from the analysis using a gating strategy based on CD19-APC-H7 (1D3) staining. T cell cycle stages were assessed using propidium iodide (PI) staining (Beckman Coulter, Roissy CDG, France) and the percentage of apoptotic T cells by Annexin V-FITC staining (BD Biosciences, Le Pont-de-Claix, France). Samples were run on a BD LSRFORTESSA X-20 (BD Biosciences, Le Pont de Claix, France), and the data were analyzed using FlowJo v10 software (FlowJo LLC, Ashland, OR, USA).

### Western Blotting

The analysis of CD3^+^CD4^+^CD25^−^ effector T cells protein expression was performed by western blotting after negative selection with MACS columns (Miltenyi Biotec. Paris, France). The following primary antibodies were used: anti-Bid, anti-actin, and anti-cleaved and total caspase 8, 9, and 12 (Cell Signaling Technology, St Quentin en Yvelines, France).

### Measurement of Mitochondrial Membrane Potential

Mitochondrial membrane potential was measured using the potential-sensitive fluorescent probe tetraethylbenzimidazolylcarbocyanine iodide (JC-1) (Life Technologies, Saint-Aubin, France). Cells were incubated in Hank's Balanced Salt Solution (Gibco Life Technologies, Saint-Aubin, France) with JC-1 at 5 mg/mL for 30 min at 37°C. CD19-APC-H7 (1D3) antibody staining allowed the removal of B cells from the analysis. Samples were assessed on a BD LSRFORTESSA X-20 (BD Biosciences, Le Pont de Claix, France), and the data were analyzed using FlowJo v10 software (FlowJo LLC, Ashland, OR, USA).

### Measurement of Mitogen-Activated Protein Kinase (MAPK) Phosphorylation

After 48 h of co-culture, cells were stained with the following Phosflow antibodies: anti-phospho-p38-PeCy-7 and anti-phospho-JNK-PE (BD Biosciences, Le Pont de Claix, France); anti-phospho-ERK1/2-PE (eBioscience, Paris, France); and CD19-APC-H7 (1D3) antibody to remove B cells from the analysis. Samples were assessed on a BD LSRFORTESSA X-20 (BD Biosciences, Le Pont de Claix, France), and the data were analyzed using FlowJo v10 software (FlowJo LLC, Ashland, OR, USA).

### Statistical Analyses

Comparisons of experimental values between the two groups were analyzed using the Mann–Whitney *U*-test. The non-parametric Kruskal–Wallis test with Dunn's posttest were used for comparisons between more than two groups. All statistical analyses were performed using GraphPad Prism v7 (La Jolla, CA, USA).

## Results

### Severe Asthmatic Patients and Asthmatic Mice Harbor a Defect in CD19^+^CD9^+^ B Cell Frequency

**Figure 1:**
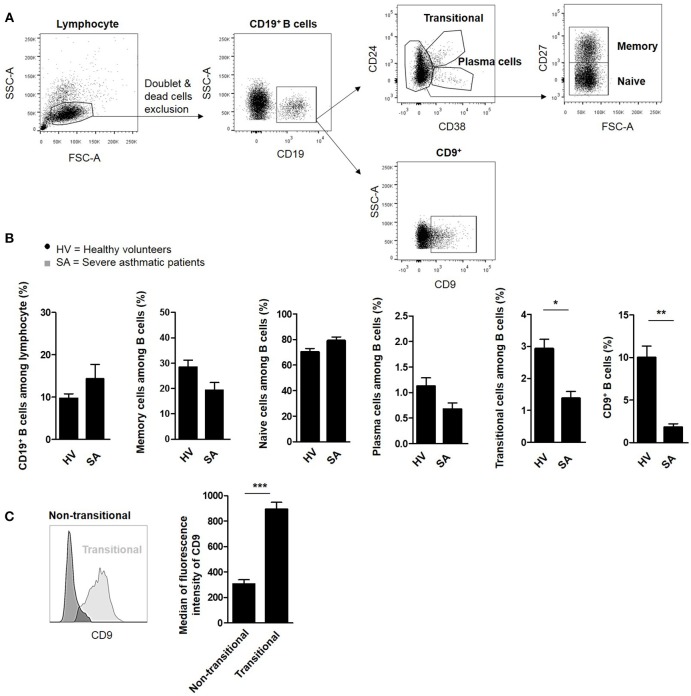
B lymphocyte subpopulations in the blood of severe asthmatic patients. **(A)** Gating strategy used after immunostaining to determine all B cell subsets. **(B)** Assessment of CD19^+^ B lymphocytes, CD19^+^CD27^+^ memory cells, CD19^+^CD27^−^ naive cells, CD19^+^CD24^−^CD38^+^ plasma cells, CD19^+^CD24^hi^CD38^hi^ transitional cells, and CD9^+^ B cells in 10 healthy volunteers (HV) and 9 severe asthmatic patients (SA) (*p < 0.05, **p < 0.01). **(C)** Expression of the mean fluorescence intensity of CD9 in transitional and non-transitional B cell subsets (***p < 0.001).

The frequencies of CD19^+^ B lymphocytes, CD19^+^CD27^+^ memory cells, CD19^+^CD27^−^ naïve cells, CD19^+^CD24^hi^CD38^hi^ transitional cells, CD19^+^CD24^−^CD38^+^ plasma cells, and CD19^+^CD9^+^ B cells were assessed by flow cytometry using PBMCs from severe asthmatic patients and HV as controls (gating strategy shown in Figure 1A). The total CD19^+^ B cell frequency was not significantly different between HV and severe asthmatic patients (Figure 1B), suggesting the absence of a systemic effect of the treatment. No significant differences were observed for naive, memory and, plasma cell subtypes between the two groups. The frequencies of CD19^+^CD24^hi^CD38^hi^ transitional and CD19^+^CD9^+^ B cells, however, were significantly lower in asthmatic patients than in HV (2.9% ± 0.3 and 9.9% ± 1.3 vs. 1.3% ± 0.2 and 1.8% ± 0.3 for HV and asthmatic patients, respectively, *p* < 0.05 and *p* < 0.01). Interestingly, all CD19^+^CD24^hi^CD38^hi^ transitional B cells expressed CD9 (median fluorescence intensity of CD9 306% ± 34 vs. 894% ± 52 in non-transitional and transitional cells, respectively, *p* < 0.001) (Figure 1C), showing that CD19^+^CD24^hi^CD38^hi^ transitional cells were included in the CD9^+^ B cell subset.

**Figure 2:**
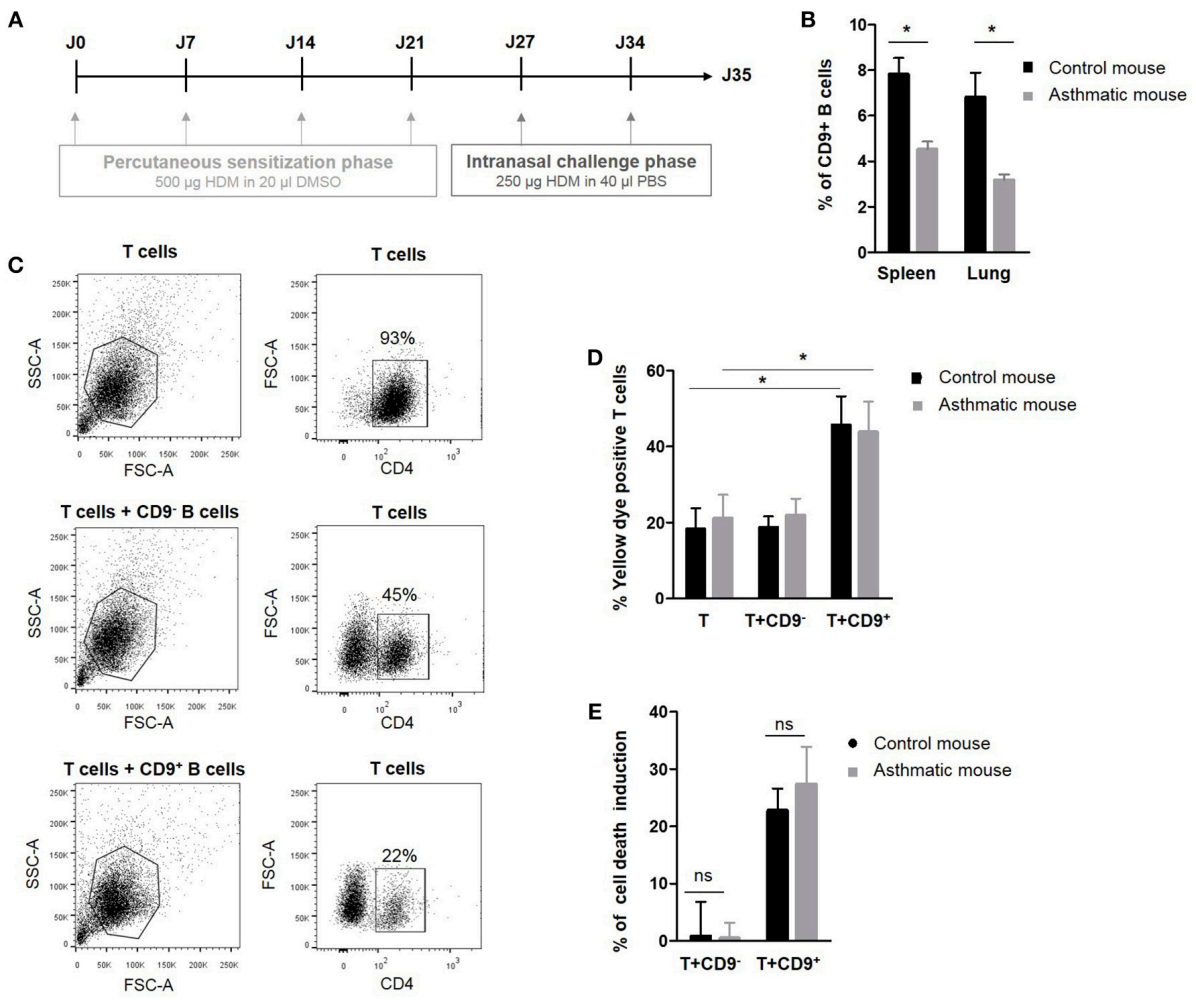
Percentage and regulatory properties of CD9^+^ B cells in asthmatic mice. **(A)** Induction protocol in asthma mice: House dust mite model. **(B)** Percentage of CD9^+^ B cells among CD19^+^ cells in the spleen and lung of control and asthmatic mice (*n* = 4, *p < 0.05). **(C)** Gating strategy used to remove B cells from the analysis by CD4 FITC staining. **(D)** After 48 h of activation, splenic CD3^+^CD4^+^CD25^−^ effector T cells from asthmatic and naive Balb-c mice were co-cultured for 48 h with CD19^+^CD9^+^ or CD19^+^CD9^−^ B cells or alone as controls. Cells were stained with yellow dye to measure T cell death induced by CD9^+^ or CD9^−^ B cells. Percentage of Annexin V-positive T cell staining (*n* = 6, *p < 0.05). **(E)** Percentage of T cell death induction by CD19^+^CD9^+^ or CD19^+^CD9^−^ B cells (ns, non-significant).

We have previously demonstrated that murine IL-10^+^ Bregs are enriched in a CD9^+^ B cell subset and that adoptive transfer of CD9^+^ B cells alone is sufficient to abrogate asthma in an IL-10-dependent manner (24). To decipher the regulatory potential of CD19^+^CD9^+^ B cells under inflammatory conditions, allergic asthma was induced in a mouse model using HDM as previously described (31) and summarized in Figure 2A. The percentage of CD19^+^CD9^+^ B cells was estimated in the spleen and lung of control and asthmatic mice using flow cytometry (Figure 2B). Asthmatic mice had significantly fewer CD19^+^CD9^+^ B cells in the spleen and lung than control mice (4.5% ± 0.3 and 3.1% ± 0.2 vs. 7.8% ± 0.7 and 6.8% ± 1 in the spleen and lung of asthmatic and control mice, respectively, *p* < 0.05). These data validate the mouse as a relevant model for asthma in humans. All together, we report that patients with severe asthma and asthmatic mice both harbor a defect in number of CD19^+^CD9^+^ B cells.

### CD19^+^CD9^+^ B Cells From Asthmatic Mice Harbor no Suppressive Property Defects

The next step was to analyze the regulatory function of CD19^+^CD9^+^ B cells in normal and pathologic situations. Thus, we analyzed the effects of CD19^+^CD9^+^ B cells from asthmatic and wild type control mice on CD3^+^CD4^+^CD25^−^ effector T cell death in co-cultures. To achieve this goal, splenic CD19^+^CD9^−^ or CD19^+^CD9^+^ B cells were activated for 48 h with anti-CD40/LPS. CD3^+^CD4^+^CD25^−^ effector T cells were activated for 48 h with IL-2. CD19^+^CD9^−^ or CD19^+^CD9^+^ B cells were then co-cultured for 48 h with CD3^+^CD4^+^CD25^−^ effector T cells at a 1:1 ratio, and cell death was measured using yellow dye staining (Figure 2C). CD19^+^CD9^+^ B cells from asthmatic mice or controls both induced CD3^+^CD4^+^CD25^−^ effector T cell death (18.2% ± 5.5 vs. 45.6% ± 7.6 in T cells alone or co-cultured with CD19^+^CD9^+^ B cells, respectively, in control mice, *p* < 0.01; 21% ± 6.2 vs. 43.8% ± 8 in T cells alone or co-cultured with CD19^+^CD9^+^ B cells, respectively, in asthmatic mice, *p* < 0.01) (Figure 2D). Moreover, the percentages of CD3^+^CD4^+^CD25^−^ effector T cell death induced by CD19^+^CD9^+^ B cells from asthmatic mice or controls were the same (22.7% ± 3.8 vs. 27.3% ± 6.5 in control and asthmatic mice, respectively, non-significant–ns) (Figure 2E). Finally, CD19^+^CD9^−^ B cells from asthmatic mice or controls did not induce CD3^+^CD4^+^CD25^−^ effector T cell death (0.8% ± 5.9 vs. 0.4% ± 2.6 in control and asthmatic mice, respectively, ns). These data show that although asthmatic mice have reduced number of CD19^+^CD9^+^ B cells, these cells display no defects in their capacity to induce T cell apoptosis. Although CD9 has been identified as a marker of B cells that are secreting IL-10, their full regulatory mechanisms remain unknown. Because there was no difference in function between CD19^+^CD9^+^ from naïve and asthmatic mouse, we further investigated the regulatory properties of these cells to decipher the molecular pathway leading to T cell death in naïve mice.

### CD19^+^CD9^+^ B Cells Induce CD3^+^CD4^+^CD25^−^ Effector T Cell Cycle Arrest in subG0/G1 and Cell Death

**Figure 3:**
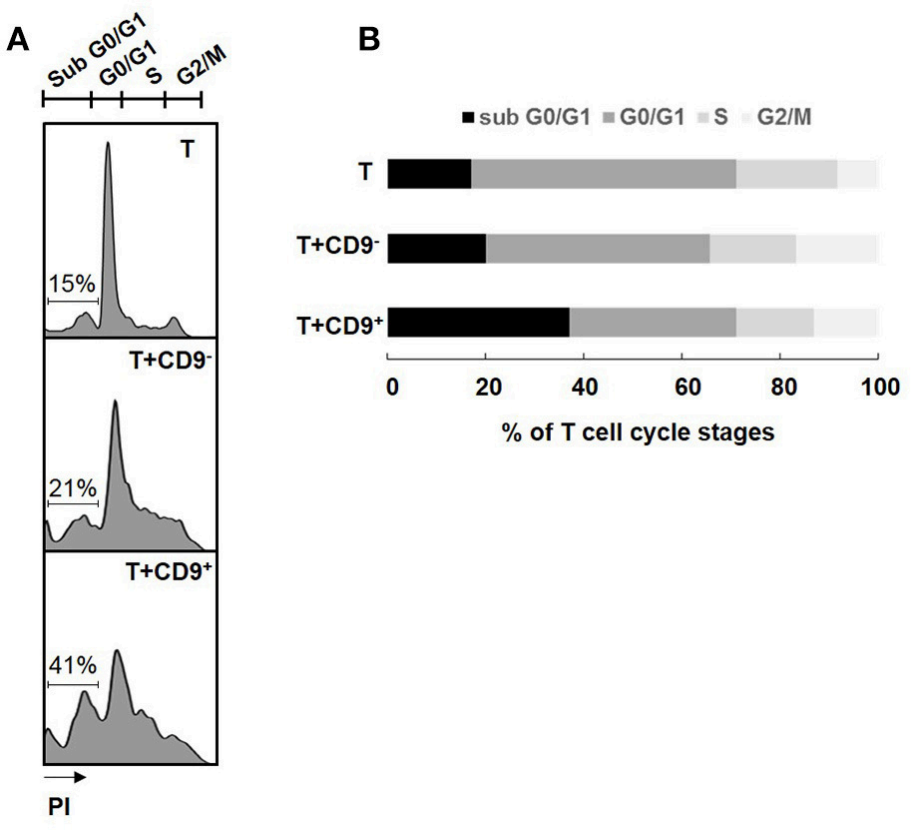
Effects of CD9^+/−^ B cells on T cell proliferation and death. After 48 h of activation, splenic CD3^+^CD4^+^CD25^−^ effector T cells from Balb-c mice were co-cultured for 48 h with CD19^+^CD9^+^ or CD19^+^CD9^−^ B cells at a 1:1 ratio or alone as controls. Cells were stained with CD4 FITC antibody to remove B cells from the analysis. **(A)** Cells were stained with propidium iodide to measure T cell cycle stages. Representative staining of the different cell cycle stages**. (B)** Percentage of T cells in the different cell cycle stages (*n* = 6).

Determination of the cell cycle stage at which a cell is stopped is a good indicator of the type of cell death induced (32). CD19^+^CD9^+^ B cells, but not CD19^+^CD9^−^ B cells, induced significant CD3^+^CD4^+^CD25^−^ effector T cell cycle arrest in sub G0/G1, as shown by PI staining (37.8% ± 4.28 and 20.2% ± 3.29 for T cells co-cultured with CD19^+^CD9^+^ B cells and T cells co-cultured with CD19^+^CD9^−^ B cells, respectively; *p* < 0.01) (Figures 3A,B). No difference was observed when T cells were co-cultured with or without CD19^+^CD9^−^ B cells. CD3^+^CD4^+^CD25^−^ effector T cell S-phase progression did not appear to be significantly altered under any of the conditions, showing that B cells had no effect on T cell proliferation *per se*. All together, these data show that CD19^+^CD9^+^ B cells, but not CD19^+^CD9^−^ B cells, induce CD3^+^CD4^+^CD25^−^ effector T cell cycle arrest in sub G0/G1, resulting in cell death.

### CD19^+^CD9^+^ B Cells Induce CD3^+^CD4^+^CD25^−^ Effector T Cell Apoptosis via an IL10-Dependent Mechanism

**Figure 4:**
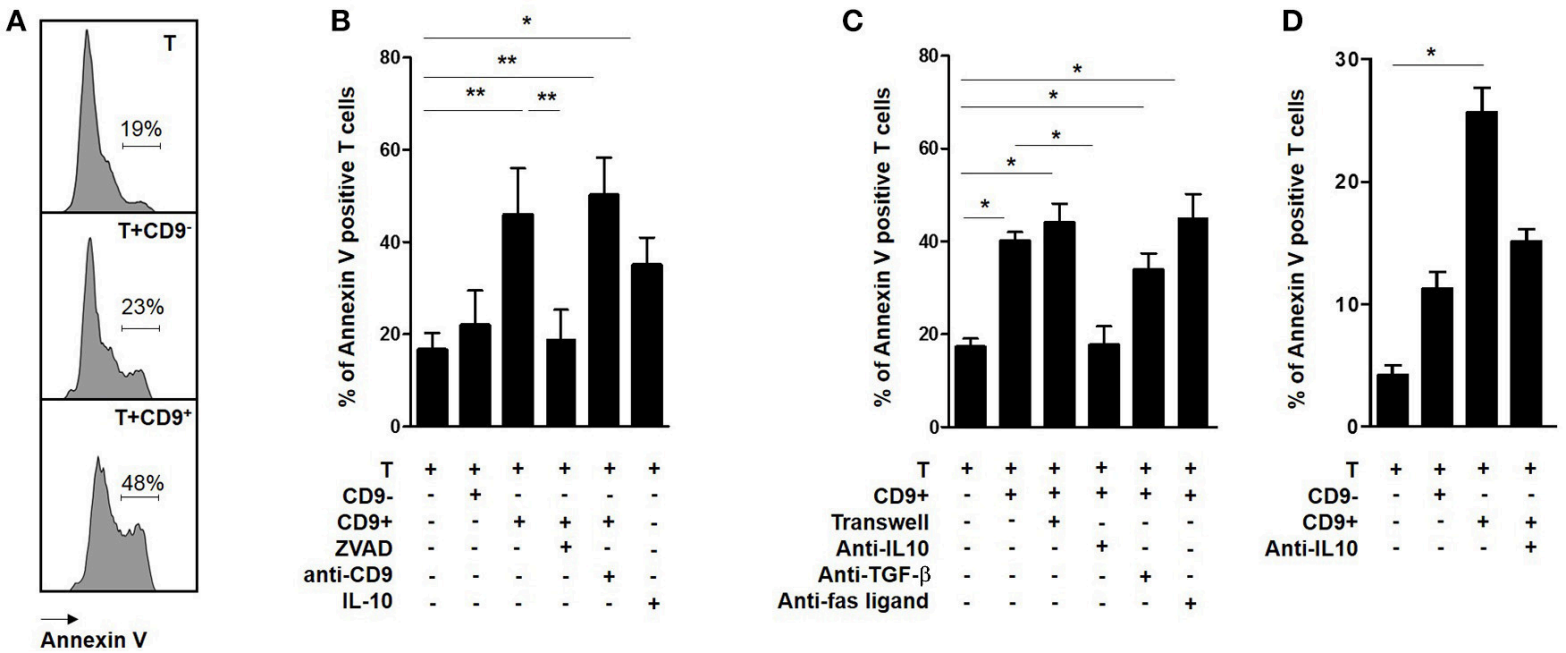
Effects of CD9^+/−^ B cells on T cell apoptosis. **(A–C)** After 48 h of activation, splenic CD3^+^CD4^+^CD25^−^ effector T cells from Balb-c mice were co-cultured for 48 h with CD19^+^CD9^+^ or CD19^+^CD9^−^ B cells or alone as controls. During co-culture, the cells were also treated with 2 μg/μL KMC8 anti-CD9 antibody, 50 nM Z-VAD, 10 ng/mL IL-10, 10 μg/mL anti–IL-10, 10 μg/mL anti–TGF-β1, or 20 μg/mL anti-Fas ligand. Cells were also co-cultured in trans-wells. The cells were stained with CD4 antibody to remove B cells from the analysis and with Annexin V to measure T cell apoptosis. **(A)** Representative results of Annexin V staining. **(B,C)** Percentage of Annexin V-positive T cells in each co-culture condition (*n* = 6, *p < 0.05, **p < 0.01). **(D)** After 48 h of activation, CD3^+^CD4^+^CD25^−^ effector T cells from 9 healthy volunteers were co-cultured for 48 h with CD19^+^CD9^+^ or CD19^+^CD9^−^ B cells or alone as controls. Cells were also treated with 20 μg/mL anti–IL-10 and stained with CD4 antibody to remove B cells from the analysis and stained with Annexin V to measure T cell apoptosis. Percentage of Annexin V-positive T cells in the different co-culture conditions (*n* = 9; *p < 0.05).

Cells in sub G0/G1 display sub-diploid content, which is an indicator of the DNA fragmentation characteristic of apoptotic cells (33). Activated CD3^+^CD4^+^CD25^−^ effector T cells were co-cultured with CD19^+^CD9^+^ or CD19^+^CD9^−^ B cells for 48 h and then stained with Annexin V (Figure 4A). The percentage of Annexin V-positive CD3^+^CD4^+^CD25^−^ effector T cells was significantly higher following co-culture with CD19^+^CD9^+^ B cells than that of T cells alone (42.1% ± 5.5 vs. 18.6% ± 3.3, respectively; *p* < 0.001), whereas no difference was observed for T cells co-cultured with CD19^+^CD9^−^ B cells (21% ± 6.5; ns) (Figure 4B). Treatment with an anti-CD9 agonist had no effect on the T cell death induced by CD19^+^CD9^+^ B cells, showing that this effect was not mediated by CD9 (50.3% ± 4.6; ns compared with T cells co-cultured with CD19^+^CD9^+^ B cells). Treatment with Z-VAD, a specific inhibitor of apoptotic cell death, blocked the T cell apoptosis induced by CD19^+^CD9^+^ B cells (18.8% ± 5.6; *p* < 0.01). Taken together, these data show that CD19^+^CD9^+^ B cells induce CD3^+^CD4^+^CD25^−^ effector T cell cycle arrest in sub G0/G1, leading to apoptosis.

To determine whether direct B cell-T cell contact was necessary for CD19^+^CD9^+^ B cells to induce T cell apoptosis, CD3^+^CD4^+^CD25^−^ effector T cells were co-cultured with CD19^+^CD9^+^ B cells in classical or trans-well plate assays (Figure 4C). CD19^+^CD9^+^ B cells induced T cell death in both situations (44% ± 4.1 vs. 40% ± 2, respectively, ns). Moreover, the T cell death induced by CD19^+^CD9^+^ B cells was not affected when the co-culture was performed in the presence of anti-TGF-β (40% ± 2 vs. 33.8% ± 3.6 with or without anti-TGF-β, respectively, ns) nor anti-Fas ligand (45% ± 5.1, ns compared with T cells co-cultured with CD19^+^CD9^+^ B cells). In contrast, anti-IL-10 prevented T cell death and fully restored T cell viability (17.2% ± 1.9 vs. 17.6% ± 4.1 for T cells alone and T cells co-cultured with CD19^+^CD9^+^ B cells/anti-IL-10, respectively, ns). Interestingly, CD3^+^CD4^+^CD25^−^ effector T cells treated with IL-10 underwent apoptosis (35% ± 3.4 *p* < 0.05 compared with T cells alone), confirming its involvement in T cell apoptosis induced by CD19^+^CD9^+^ B cells. In summary, these data show that T cell-B cell contact is not necessary for T cell apoptosis induction by CD19^+^CD9^+^ B cells and that apoptosis induction is dependent on IL-10.

To determine whether the regulatory molecular pathways of CD19^+^CD9^+^ B cells is similar in humans, CD19^+^CD9^−^ B cells, CD19^+^CD9^+^ B cells, and CD3^+^CD4^+^CD25^−^ effector T cells were sorted from the PBMCs of HV. Cells were activated for 48 h (anti-CD40 + CpG for CD19^+^CD9^−^ and CD19^+^CD9^+^B cells and IL-2 for CD3^+^CD4^+^CD25^−^ effector T cells). CD3^+^CD4^+^CD25^−^ effector T cells were co-cultured for 48 h with CD19^+^CD9^−^ B cells or CD19^+^CD9^+^ B cells at ratios of 1:1 or alone as controls. The percentage of apoptotic T cells was analyzed by Annexin V staining (Figure 4D). As for the mice, only human CD19^+^CD9^+^ B cells, but not CD19^+^CD9^−^ B cells, induced apoptosis of T cells (2.8% ± 0.8 for T cells alone, 14.3% ± 1.7 for T cells co-cultured with CD19^+^CD9^−^ B cells, and 21.4% ± 2.7 for T cells co-cultured with CD19^+^CD9^+^ B cells; T cells alone vs. T cells co-cultured with CD19^+^CD9^+^ B cells, *p* < 0.001). Treatment with anti-IL-10 reduced apoptosis induced by human T cells (15.7% ± 1.1; T cells alone vs. T cells co-cultured with CD19^+^CD9^+^ B cells/anti-IL-10, ns). Taken together, these data show that, as in mice, human CD19^+^CD9^+^ B cells induce CD3^+^CD4^+^CD25^−^ effector T cell apoptosis via IL-10.

### CD19^+^CD9^+^ B Cells Induce Apoptosis of CD3^+^CD4^+^CD25^−^ Effector T Cells Through Extrinsic and Intrinsic Apoptosis Pathways via a MAPK-Dependent Mechanism

**Figure 5:**
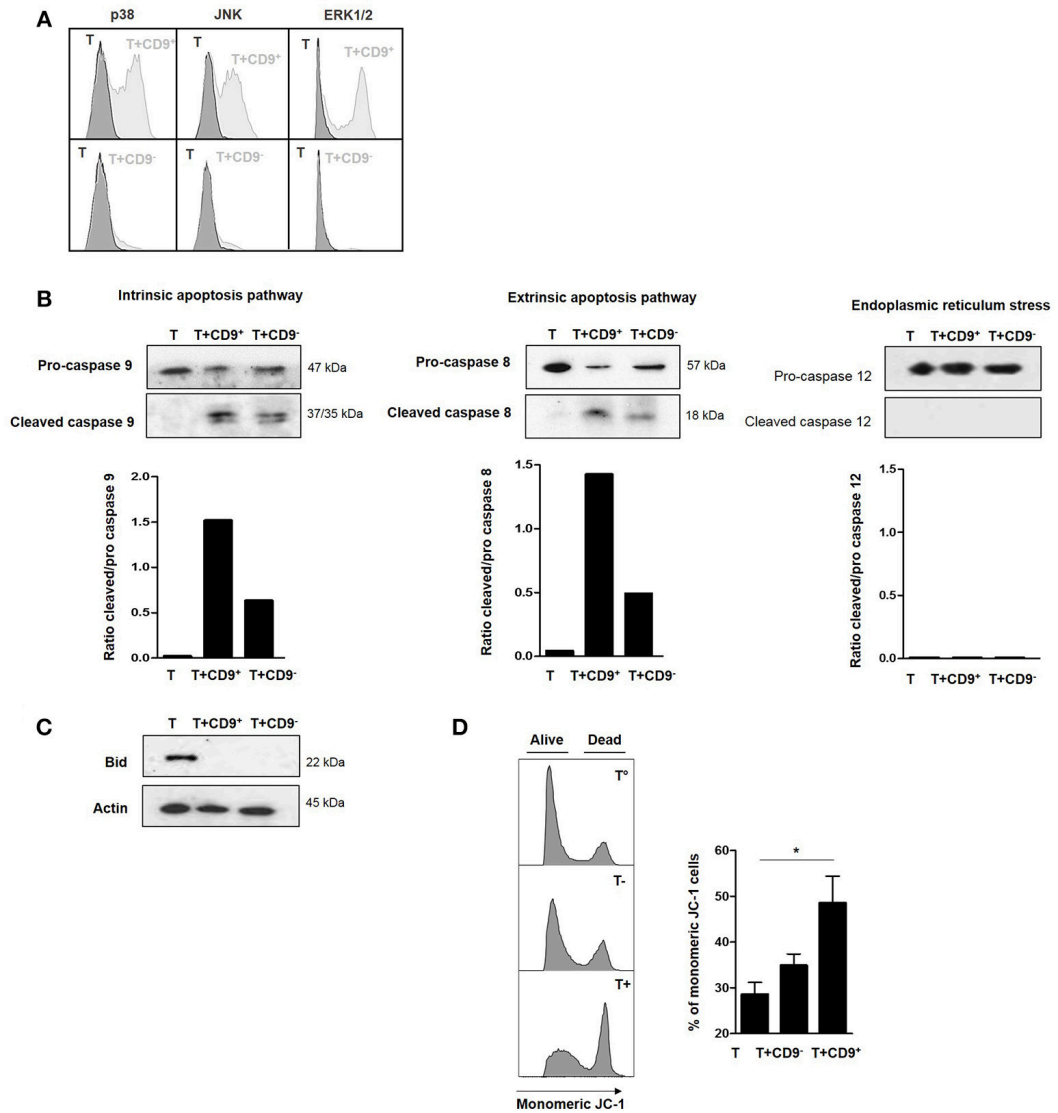
Effects of CD9^+^ B cells on MAPK phosphorylation, Bid and caspase cleavage and mitochondrial depolarization. After 48 h of activation, splenic CD3^+^CD4^+^CD25^−^ effector T cells from Balb-c mice were co-cultured for 48 h with CD19^+^CD9^+^, CD19^+^CD9^−^ B cells or alone as controls. **(A)** B cells were removed from the analysis by CD4 staining. Phosphorylation of MAPK p38, JNK, and ERK1/2 in CD3^+^CD4^+^CD25^−^ effector T cells was assessed by flow cytometry. Representative results of MAPK staining. **(B)** CD3^+^CD4^+^CD25^−^ effector T cells were negatively selected using MACS columns. Cleavage of caspase 8, 9, and 12 was assessed by western blotting, and the ratios of cleaved/pro-caspases were calculated. **(C)** CD3^+^CD4^+^CD25^−^ effector T cells were negatively selected by MACS columns. Bid cleavage was assessed by western blotting. Actin was used as the loading control. **(D)** Mitochondrial depolarization was assessed by JC-1 staining. Representative results of JC-1 staining and percentage of monomeric JC-1 cells in all culture conditions (*n* = 5, *p < 0.05).

To determine the molecular pathways that were activated by CD19^+^CD9^+^ B cells, we used flow cytometry to analyze the phosphorylation levels of MAPK p38, extracellular signal–regulated kinases (ERK1/2) and c-Jun N-terminal kinases (JNK), transcription factors that are known to be major activators of apoptosis (34, 35), in CD3^+^CD4^+^CD25^−^ effector T cells after co-culture with or without CD19^+^CD9^+^ or CD19^+^CD9^−^ B cells. Only CD19^+^CD9^+^ B cells, but not CD19^+^CD9^−^ B cells, induced phosphorylation of p38, JNK, and ERK1/2 in CD3^+^CD4^+^CD25^−^ effector T cells undergoing apoptosis (Figure 5A). Cleavage of caspases 8 and 9 is characteristic of the activation of the extrinsic and intrinsic pathways of apoptosis, respectively. Caspase 12 cleavage is characteristic of endoplasmic reticulum stress. CD3^+^CD4^+^CD25^−^ effector T cells were co-cultured with CD19^+^CD9^−^ or CD19^+^CD9^+^ B cells and negatively selected by MACS columns to perform western blotting only on T cells (purity, 94% ± 2). CD3^+^CD4^+^CD25^−^ effector T cells cultured alone were used as controls. Both caspase 8 and 9 were cleaved when CD3^+^CD4^+^CD25^−^ effector T cells were co-cultured with CD19^+^CD9^+^ B cells (Figure 5B) (ratio of cleaved/pro-caspases was 1.5 and 1.4, respectively). Caspases 8 and 9 were also cleaved when CD3^+^CD4^+^CD25^−^ effector T cells were co-cultured with CD19^+^CD9^−^ B cells, but to a reduced extent, probably due to the small percentage of Bregs not expressing CD9 (ratio of cleaved/pro-caspases was 0.7 and 0.4, respectively). Caspase 12 was not cleaved under any condition. This finding correlated with the cleavage and activation of the pro-apoptotic protein Bid, linking the intrinsic and extrinsic pathways (Figure 5C). Finally, the significantly higher percentage of monomeric JC-1 in CD3^+^CD4^+^CD25^−^ effector T cells co-cultured with CD19^+^CD9^+^ B cells confirmed the mitochondrial depolarization of T cells and activation of the intrinsic pathway of apoptosis (48.6% ± 5.7 vs. 28.6% ± 2.5, respectively; *p* < 0.05) (Figure 5D). Taken together, these data show that CD19^+^CD9^+^ B cells induce CD3^+^CD4^+^CD25^−^ effector T cell apoptosis via both extrinsic and intrinsic pathways through MAPK activation.

## Discussion

The prevalence of allergic asthma has dramatically increased worldwide during the last decade. Asthma is a chronic inflammatory disease of the airways associated with airway hyper-responsiveness to inhaled allergens and deregulation of type 2 immunity (36); however, its pathogenesis is still not fully understood. Asthma is characterized by an expansion of CD4^+^ helper T lymphocytes (Th2, Th17), increased production of the Th2 cytokines IL-4, IL-5, and IL-13, an increased level of allergen-specific immunoglobulin E (IgE), eosinophilia and inflammation of the airways (37). The roles of other T cells, including natural killer T cells, γδ T cells, and CD8 T cells, has also been reported in asthma, although the relative importance of these different cell populations remains to be confirmed (38). The role of “conventional” B cells, as an actor in immunity, has been clearly demonstrated in asthma (22, 39), and several studies in mice and humans that have also evidenced a role for “specific subsets” of B cells with regulatory properties have reported that these “Breg cells” are involved in the suppression and control of airway inflammation (6, 10). Their numbers are a crucial determinant that clearly contributes to the regulation of the immune system, and their deficiency in asthma is now well-demonstrated (21, 22, 24).

The most commonly used marker of Bregs is their ability to produce and have a suppressive effect via IL-10, but other cytokines or molecules, such as TGF-β, IL-35, and granzyme B, have been shown to mediate regulatory properties on different immune cell targets (1, 12, 14, 23), and no exclusive cell surface marker has yet been evidenced for this Breg population (5, 18). We have previously reported that asthmatic mice are characterized by a lack of IL-10-secreting Bregs enriched in a CD9^+^ B cell subset. The adoptive transfer of these CD9^+^ B cells alone is thus sufficient to abrogate asthma in an IL-10-dependent manner (24). The ability of Bregs to normalize lung function and airway inflammation and the notable alterations of this Breg pool in asthmatic mice clearly point to these cells as an interesting target in allergic diseases.

We demonstrated that these cells regulate effector T cell proliferation, but the mechanisms responsible for such an effect remain unknown. In this study, we demonstrated that CD9^+^ B cells harbored strong suppressive properties. These cells induced the death of effector T cells via the intrinsic and extrinsic pathways of apoptosis in an IL-10-dependent manner and through the MAPK cascade. We also confirmed these findings, for the first time, in humans and reported that severe asthmatic patients have reduced number of CD9^+^ B cells with regulatory properties.

CD9 is a tetraspanin molecule that is involved in different mechanisms, including adhesion, migration, cell fusion and membrane signaling (40, 41). Interestingly, CD9 is also involved in the enhancement and maintenance of IL-10 secretion in murine and human antigen-presenting cells (40, 41). We have demonstrated that an IL10^+^ B cell population is enriched in a CD9^+^ B cell subset that is decreased in asthmatic mice (24). Similar data have been published by Sun and Matsushita, who identified CD9 as a marker of murine IL-10-competent Bregs (26, 27). It has been reported that the PI3K-Akt pathway is essential for their development and is involved in their regulatory effects in a model of contact hypersensitivity (27). These data confirmed a study conducted by Ostrowski et al. in a model of foot-and-mouth disease virus-infected mice, in which CD9^+^ B cells were the main source of splenic IL-10 (25). Thus, these data not only clearly support CD9 as a potential biomarker of Bregs but also suggest a possible role in asthma physiopathology, differing from the classical role of B cells as IgE producers. However, no mechanism of action has yet been reported for CD9^+^ B cells.

The unique link between CD9 and asthma is related to the observation that certain tetraspanins interact with tetrameric high-affinity IgE receptors (FcεRI) on effector cells (42, 43). This interaction is associated with the negative regulation of FcεRI-mediated degranulation of mast cells, which may at least in part explain their possible role in asthma. We suggest herein another or complementary potential mechanism for CD9 Breg cells in this pathology. We report that CD9^+^ B cells induce T cell cycle arrest in sub G0/G1, leading to the cell death of effector T cells by apoptosis via IL-10 secretion and activation of the MAPK cascade independently from CD9 itself. Apoptosis is a physiologically programmed cell death program that is also involved in defense mechanisms against damaged or stressed cells and cells stimulated by any agent to prevent the accumulation of non-functional cells in tissues (44). Apoptosis may be activated via two different pathways: the intrinsic and extrinsic pathways (45, 46). Following the activation of different signaling cascades, both pathways converge at the final caspase 3 activation step and commonly lead to the cleavage of different proteins leading to cell death. The link between IL-10 and apoptosis is clear, and our data are concordant with those of O'Farrell's data showing that IL-10 increases the expression of p19 to inhibit macrophage proliferation through cell cycle arrest in G1 and implicating the MAPK phosphorylation cascade (47). In our experiments, IL10 alone is able to induce the apoptosis of T cells as CD9^+^ B cells. IL-10 inhibits the proliferation of other cells with various other effects, such as the induction of endoplasmic reticulum stress (48). These cells did not induce cleavage of caspase 12, which is characteristic of endoplasmic reticulum stress, nor necroptosis, as treatment with necrostatin-1 did not abrogate T cell death (data not shown), confirming the involvement of apoptosis.

The intrinsic apoptosis pathway is initiated by the activation of pro-apoptotic mediators leading to mitochondrial depolarization, the release of cytochrome c and cleavage of caspase 9 (49). The extrinsic pathway is activated when death receptors are ligated, resulting in caspase 8 activation (50). Caspase 8 can directly induce apoptosis or activate the intrinsic pathway by cleavage of Bid. Here, we report that CD9^+^ B cells induced T cell apoptosis through both the extrinsic and intrinsic pathways, involving caspases 8 and 9 and cleavage of the molecule Bid required for apoptosis activation. Activation of the intrinsic pathway in T cells by CD9^+^ Bregs was confirmed by mitochondrial depolarization. These data corroborate Bailey's results showing that IL-10 induces apoptosis in mast cells and macrophages via the intrinsic cascade through mechanisms involving caspase 3 cleavage and mitochondrial depolarization (51). Surprisingly, when T cells are co-cultured with non-regulatory CD9- B cells, low levels of caspase 8 and 9, and Bid are cleaved. These phenomena could explain the small but substantial number of apoptotic cells compared with T cells alone. This low level of T cell death is more likely a consequence of CD9 as a known marker of enrichment of IL-10-secreting B cells (88% of total IL-10 secreting B cells) (26) and the presence of a few Breg cells in the CD9^−^ subset.

We have also confirmed that CD9^+^ B cells also exist in humans. CD9^+^ B cells from HV induced apoptosis of autologous effector T cells in an IL-10-dependent manner, as observed for mouse CD9^+^ B cells. Finally, we report for the first time that CD9^+^ B cells, which are also associated with a CD19^+^CD24^hi^CD38^hi^ transitional B cell phenotype, were reduced in number in the blood of severe asthmatic patients. There is evidence in different models that enhanced activity of Bregs producing IL-10 strongly suppresses inflammatory processes (4–7, 20), and conversely, Van Der Vlugt reported that patients with allergic asthma displayed B cells with impaired regulatory activity (21). Here, we report that the number of CD9^+^ B cells was reduced in the blood of severe asthmatic patients, as demonstrated in asthmatic mice lacking CD9^+^ B cells but displaying no functional defect. Furthermore, these cells were able to induce effector T cell apoptosis in the same manner as CD9^+^ B cells from wild type mice.

Interestingly, we also report herein that severe asthmatic patients had fewer CD9^+^ B cells not only at basal levels between exacerbations but also following acute inflammation events (data not shown), suggesting that even under a strong inflammatory environment, patients are not able to upregulate CD9^+^ Breg production. These data suggest that the restoration of a normal number of CD9^+^ B cells may “correct” a potential defect and suggest that increasing Breg cells in allergic/asthmatic patients may be envisioned as a new therapeutic strategy. In mice, the adoptive transfer of CD9^+^ B cells reverses asthma (24), and the adoptive transfer of CD1d^hi^ Bregs suppresses airway inflammation by inducing natural forkhead box protein 3-positive CD4+ regulatory T cell recruitment into the lungs (10). Interestingly, parasites and infectious agents drive Breg expansion and have been proposed as a potential mechanism for reducing host immune responses (19, 52–54). The potential for use of helminth molecules as a new-anti-inflammatory therapy has been postulated using the worm's molecules to generate Breg cells (54, 55). Modulation of anti-inflammatory cytokines (56), the possibility to expand Breg cells *in vitro* (9, 57) or strategies aimed at controlling signaling pathways in favor of *in vivo* Breg expansion (58) have been seriously considered. Compared with allergen-specific immunotherapy or non-specific treatments, such strategies may offer the possibility for a reduced treatment time and superior effects resulting from self-sustaining or the induction of regulatory mechanisms since targeting Breg cells may also embrace other regulatory systems simultaneously, such as IL-10-producing regulatory T cells (19).

In conclusion, our data are the first to characterize the molecular mechanisms of the regulation of CD9^+^ B cells to induce effector T cell apoptosis in mice and humans. The lack of CD9^+^ Breg cells in severe asthmatic patients suggests Bregs as a potential target for future therapeutic strategies. A better understanding of the immunological mechanisms underlying allergic diseases is essential for the development of new preventive and therapeutic Breg interventions.

## Author Contributions

CB, MD, LuC, GB, SB, and AM: study conception and design. CB, MD, and ED: acquisition of data. CB, MD, LuC, GB, SB, and AM: analysis and interpretation of data. All authors critical revision for important intellectual content. CB and MD: statistical analysis. CB, MD, AM, and SB: obtained funding. ED, AF, M-AC, LuC, and MK: administrative, technical, or material support. SB and AM: carried out study supervision. All authors approved the final version of the manuscript prior to submission.

### Conflict of Interest Statement

The authors declare that the research was conducted in the absence of any commercial or financial relationships that could be construed as a potential conflict of interest.

## References

[1] Fillatreau S. Cytokine-producing B cells as regulators of pathogenic and protective immune responses. Ann Rheum Dis. (2013) 72(Suppl. 2):ii80–4. 10.1136/annrheumdis-2012-202253 23253921

[2] Crow MK. Costimulatory molecules and T-cell-B-cell interactions. Rheum Dis Clin North Am. (2004) 30:175–91, vii–viii. 10.1016/S0889-857X(03)00111-X 15061574

[3] Zanetti M Castiglioni P Rizzi M Wheeler M Gerloni MB. Lymphocytes as antigen-presenting cell-based genetic vaccines. Immunol Rev. (2004) 199:264–78. 10.1111/j.0105-2896.2004.00152.x 15233740

[4] Lund FE Randall TD. Effector and regulatory B cells: modulators of CD4+ T cell immunity. Nat Rev Immunol. (2010) 10:236–47. 10.1038/nri2729 20224569 PMC3038334

[5] Rosser EC Mauri C. Regulatory B cells: origin, phenotype, and function. Immunity (2015) 42:607–12. 10.1016/j.immuni.2015.04.005 25902480

[6] van de Veen W Stanic B Wirz OF Jansen K Globinska A Akdis M Role of regulatory B cells in immune tolerance to allergens and beyond. J Allergy Clin Immunol. (2016) 138:654–65. 10.1016/j.jaci.2016.07.006 27596706

[7] Fillatreau S Sweenie CH McGeachy MJ Gray D Anderton SM. B cells regulate autoimmunity by provision of IL-10. Nat Immunol. (2002) 3:944–50. 10.1038/ni833 12244307

[8] Ray A Dittel BN. Mechanisms of regulatory B cell function in autoimmune and inflammatory diseases beyond IL-10. J Clin Med. (2017) 6:12. 10.3390/jcm6010012 28124981 PMC5294965

[9] Blair PA Chavez-Rueda KA Evans JG Shlomchik MJ Eddaoudi A Isenberg DA. Selective targeting of B cells with agonistic anti-CD40 is an efficacious strategy for the generation of induced regulatory T2-like B cells and for the suppression of lupus in MRL/lpr mice. J Immunol. (2009) 182:3492–502. 10.4049/jimmunol.0803052 19265127 PMC4082659

[10] Amu S Saunders SP Kronenberg M Mangan NE Atzberger A Fallon PG. Regulatory B cells prevent and reverse allergic airway inflammation via FoxP3-positive T regulatory cells in a murine model. J Allergy Clin Immunol. (2010) 125:1114–24.e8. 10.1016/j.jaci.2010.01.018 20304473

[11] Mauri C Gray D Mushtaq N Londei M. Prevention of arthritis by interleukin 10-producing B cells. J Exp Med. (2003) 197:489–501. 10.1084/jem.20021293 12591906 PMC2193864

[12] Cherukuri A Rothstein DM Clark B Carter CR Davison A Hernandez-Fuentes M. Immunologic human renal allograft injury associates with an altered IL-10/TNF-α expression ratio in regulatory B cells. J Am Soc Nephrol. (2014) 25:1575–85. 10.1681/ASN.2013080837 24610932 PMC4073434

[13] Nouël A Simon Q Jamin C Pers J-O Hillion S. Regulatory B cells: an exciting target for future therapeutics in transplantation. Front Immunol. (2014) 5:11. 10.3389/fimmu.2014.00011 24478776 PMC3897876

[14] Chesneau M Michel L Dugast E Chenouard A Baron D Pallier A. tolerant kidney transplant patients produce B cells with regulatory properties. J Am Soc Nephrol. (2015) 26:2588–98. 10.1681/ASN.2014040404 25644114 PMC4587683

[15] Shabir S Girdlestone J Briggs D Kaul B Smith H Daga S. Transitional B lymphocytes are associated with protection from kidney allograft rejection: a prospective study. Am J Transplant. (2015) 15:1384–91. 10.1111/ajt.13122 25808898

[16] Khoder A Sarvaria A Alsuliman A Chew C Sekine T Cooper N. Regulatory B cells are enriched within the IgM memory and transitional subsets in healthy donors but are deficient in chronic GVHD. Blood (2014) 124:2034–45. 10.1182/blood-2014-04-571125 25051962 PMC4186534

[17] Chong AS Khiew SH. Transplantation tolerance: don't forget about the B cells. Clin Exp Immunol. (2017) 189:171–80. 10.1111/cei.12927 28100001 PMC5508319

[18] Mauri C Menon M. The expanding family of regulatory B cells. Int Immunol. (2015) 27:479–86. 10.1093/intimm/dxv038 26071023 PMC4587489

[19] van der Vlugt LEPM Labuda LA Ozir-Fazalalikhan A Lievers E Gloudemans AK Liu K-Y Schistosomes induce regulatory features in human and mouse CD1d(hi) B cells: inhibition of allergic inflammation by IL-10 and regulatory T cells. PLoS ONE (2012) 7:e30883 10.1371/journal.pone.0030883 22347409 PMC3275567

[20] Noh J Lee JH Noh G Bang SY Kim HS Choi WS. Characterisation of allergen-specific responses of IL-10-producing regulatory B cells (Br1) in Cow Milk Allergy. Cell Immunol. (2010) 264:143–9. 10.1016/j.cellimm.2010.05.013 20646682

[21] van der Vlugt LEPM Mlejnek E Ozir-Fazalalikhan A Janssen Bonas M Dijksman TR Labuda LA CD24(hi)CD27(+) B cells from patients with allergic asthma have impaired regulatory activity in response to lipopolysaccharide. Clin Exp Allergy (2014) 44:517–28. 10.1111/cea.12238 24261983

[22] Kamekura R Shigehara K Miyajima S Jitsukawa S Kawata K Yamashita K. Alteration of circulating type 2 follicular helper T cells and regulatory B cells underlies the comorbid association of allergic rhinitis with bronchial asthma. Clin Immunol. (2015) 158:204–11. 10.1016/j.clim.2015.02.016 25829231

[23] Braza F Chesne J Castagnet S Magnan A Brouard S. Regulatory functions of B cells in allergic diseases. Allergy (2014) 69:1454–63. 10.1111/all.12490 25060230

[24] Braza F Chesne J Durand M Dirou S Brosseau C Mahay G. A regulatory CD9(+) B-cell subset inhibits HDM-induced allergic airway inflammation. Allergy (2015) 70:1421–31. 10.1111/all.12697 26194936

[25] Ostrowski M Vermeulen M Zabal O Zamorano PI Sadir AM Geffner JR. The early protective thymus-independent antibody response to foot-and-mouth disease virus is mediated by splenic CD9+ B lymphocytes. J Virol. (2007) 81:9357–67. 10.1128/JVI.00677-07 17567692 PMC1951431

[26] Sun J Wang J Pefanis E Chao J Rothschild G Tachibana I. Transcriptomics identify CD9 as a marker of murine IL-10-competent regulatory B cells. Cell Rep. (2015) 13:1110–7. 10.1016/j.celrep.2015.09.070 26527007 PMC4644501

[27] Matsushita T Le Huu D Kobayashi T Hamaguchi Y Hasegawa M Naka K. A novel splenic B1 regulatory cell subset suppresses allergic disease through phosphatidylinositol 3-kinase-Akt pathway activation. J Allergy Clin Immunol. (2016) 138:1170–82.e9. 10.1016/j.jaci.2015.12.1319 26948079

[28] Proceedings of the ATS workshop on refractory asthma: current understanding recommendations and unanswered questions American thoracic society. Am J Respir Crit Care Med. (2000) 162:2341–51. 10.1164/ajrccm.162.6.ats9-00 11112161

[29] Quanjer PH Stanojevic S Cole TJ Baur X Hall GL Culver BH. Multi-ethnic reference values for spirometry for the 3-95-yr age range: the global lung function 2012 equations. Eur Respir J. (2012) 40:1324–43. 10.1183/09031936.00080312 22743675 PMC3786581

[30] Chung KF Wenzel SE Brozek JL Bush A Castro M Sterk PJ. International ERS/ATS guidelines on definition, evaluation and treatment of severe asthma. Eur Respir J. (2014) 43:343–73. 10.1183/13993003.52020-2013 24337046

[31] Chesné J Braza F Chadeuf G Mahay G Cheminant M-A Loy J. Prime role of IL-17A in neutrophilia and airway smooth muscle contraction in a house dust mite-induced allergic asthma model. J Allergy Clin Immunol. (2015) 135:1643–1643.e3. 10.1016/j.jaci.2014.12.1872 25649077

[32] Surova O Zhivotovsky B. Various modes of cell death induced by DNA damage. Oncogene (2013) 32:3789–97. 10.1038/onc.2012.556 23208502

[33] Crowley LC Chojnowski G Waterhouse NJ. Measuring the DNA content of cells in apoptosis and at different cell-cycle stages by propidium iodide staining and flow cytometry. Cold Spring Harb Protoc. (2016) 2016:905–10. 10.1101/pdb.prot087247 27698234

[34] Brosseau CM Pirianov G Colston KW. Involvement of stress activated protein kinases (JNK and p38) in 1,25 dihydroxyvitamin D3-induced breast cell death. Steroids (2010) 75:1082–8. 10.1016/j.steroids.2010.07.007 20654640

[35] Wada T Penninger JM. Mitogen-activated protein kinases in apoptosis regulation. Oncogene (2004) 23:2838–49. 10.1038/sj.onc.1207556 15077147

[36] Edwards MR Saglani S Schwarze J Skevaki C Smith JA Ainsworth B. Addressing unmet needs in understanding asthma mechanisms: from the European asthma research and innovation partnership (EARIP) work package (WP)2 collaborators. Eur Respir J. (2017) 49:1602448. 10.1183/13993003.02448-2016 28461300

[37] Akdis M Akdis CA. Mechanisms of allergen-specific immunotherapy: multiple suppressor factors at work in immune tolerance to allergens. J Allergy Clin Immunol. (2014) 133:621–31. 10.1016/j.jaci.2013.12.1088 24581429

[38] Robinson DS. The role of the T cell in asthma. J Allergy Clin Immunol. (2010) 126:1081–91; quiz 1092–3. 10.1016/j.jaci.2010.06.025 20709383

[39] Lundy SK Berlin AA Martens TF Lukacs NW. Deficiency of regulatory B cells increases allergic airway inflammation. Inflamm Res. (2005) 54:514–21. 10.1007/s00011-005-1387-0 16389573 PMC3533497

[40] Peng WM Yu CF Kolanus W Mazzocca A Bieber T Kraft S Tetraspanins CD9 and CD81 are molecular partners of trimeric Fc€ RI on human antigen-presenting cells. Allergy (2011) 66:605–611. 10.1111/j.1398-9995.2010.02524.x 21241315

[41] Ha CT Waterhouse R Wessells J Wu JA Dveksler GS. Binding of pregnancy-specific glycoprotein 17 to CD9 on macrophages induces secretion of IL-10, IL-6, PGE2, and TGF-beta1. J Leukoc Biol. (2005) 77:948–57. 10.1189/jlb.0804453 15772125

[42] Higginbottom A Wilkinson I McCullough B Lanza F Azorsa DO Partridge LJ. Antibody cross-linking of human CD9 and the high-affinity immunoglobulin E receptor stimulates secretion from transfected rat basophilic leukaemia cells. Immunology (2000) 99:546–52. 10.1046/j.1365-2567.2000.00992.x 10792502 PMC2327194

[43] Moseley GW. Tetraspanin-Fc receptor interactions. Platelets (2005) 16:3–12. 10.1080/09537100400004363 15763890

[44] Dillon CP Green DR. Molecular cell biology of apoptosis and necroptosis in cancer. Adv Exp Med Biol. (2016) 930:1–23. 10.1007/978-3-319-39406-0_1 27558815

[45] Czabotar PE Lessene G Strasser A Adams JM. Control of apoptosis by the BCL-2 protein family: implications for physiology and therapy. Nat Rev Mol Cell Biol. (2014) 15:49–63. 10.1038/nrm3722 24355989

[46] Brosseau C Dousset C Touzeau C Maïga S Moreau P Amiot M. Combination of lenalidomide with vitamin D3 induces apoptosis in mantle cell lymphoma via demethylation of BIK. Cell Death Dis. (2014) 5:e1389. 10.1038/cddis.2014.346 25165875 PMC4454319

[47] O'Farrell AM Parry DA Zindy F Roussel MF Lees E Moore KW. Stat3-dependent induction of p19INK4D by IL-10 contributes to inhibition of macrophage proliferation. J Immunol. (2000) 164:4607–15. 10.4049/jimmunol.164.9.4607 10779764

[48] Lee T-S Chau L-Y. Heme oxygenase-1 mediates the anti-inflammatory effect of interleukin-10 in mice. Nat Med. (2002) 8:240–6. 10.1038/nm0302-240 11875494

[49] Brunelle JK Letai A. Control of mitochondrial apoptosis by the Bcl-2 family. J Cell Sci. (2009) 122:437–41. 10.1242/jcs.031682 19193868 PMC2714431

[50] Tait SWG Green DR. Mitochondria and cell death: outer membrane permeabilization and beyond. Nat Rev Mol Cell Biol. (2010) 11:621–32. 10.1038/nrm2952 20683470

[51] Bailey DP Kashyap M Bouton LA Murray PJ Ryan JJ. Interleukin-10 induces apoptosis in developing mast cells and macrophages. J Leukoc Biol. (2006) 80:581–9. 10.1189/jlb.0405201 16829633

[52] Hussaarts L van der Vlugt LEPM Yazdanbakhsh M Smits HH. Regulatory B-cell induction by helminths: implications for allergic disease. J Allergy Clin Immunol. (2011) 128:733–9. 10.1016/j.jaci.2011.05.012 21684587

[53] Tian F Hu X Xian K Zong D Liu H Wei H. B10 cells induced by Schistosoma japonicum soluble egg antigens modulated regulatory T cells and cytokine production of T cells. Parasitol Res. (2015) 114:3827–34. 10.1007/s00436-015-4613-x 26149531

[54] Haeberlein S Obieglo K Ozir-Fazalalikhan A Chayé MAM Veninga H van der Vlugt LEPM. Schistosome egg antigens, including the glycoprotein IPSE/alpha-1, trigger the development of regulatory B cells. PLoS Pathog. (2017) 13:e1006539. 10.1371/journal.ppat.1006539 28753651 PMC5550006

[55] Fallon PG Alcami A. Pathogen-derived immunomodulatory molecules: future immunotherapeutics? Trends Immunol. (2006) 27:470–6. 10.1016/j.it.2006.08.002 16920025

[56] Dong J Wong CK Cai Z Jiao D Chu M Lam CWK. Amelioration of allergic airway inflammation in mice by regulatory IL-35 through dampening inflammatory dendritic cells. Allergy (2015) 70:921–32. 10.1111/all.12631 25869299

[57] Chesneau M Pallier A Braza F Lacombe G Le Gallou S Baron D. Unique B cell differentiation profile in tolerant kidney transplant patients. Am J Transplant. (2014) 14:144–55. 10.1111/ajt.12508 24354874

[58] Korczak-Kowalska G Stelmaszczyk-Emmel A Bocian K Kiernozek E Drela N Domagała-Kulawik J. expanding diversity and common goal ofregulatory T and B cells. II: in allergy, malignancy, and transplantation. Arch Immunol Ther Exp. (2017) 65:523–35. 10.1007/s00005-017-0471-9 28470464 PMC5688211

