# Supplementary material for: CD9+ Regulatory B Cells Induce T Cell Apoptosis via IL-10 and Are Reduced in Severe Asthmatic Patients
Source: Front Immunol. 2018 Dec 21;9:3034. doi: 10.3389/fimmu.2018.03034 (PMC6308143; doi:10.3389/fimmu.2018.03034)
Supplement: Supplementary file 1 [file Data_Sheet_1.PDF]

**Table 1: Demographic and clinical data.**

*Definition of abbreviations:* HV, healthy volunteers; SA, severe asthmatic patients, FEV1, Forced Expiratory Volume in the first second; ACQ, asthma control questionnaire, NA, not applicable.

| | **HV** | **SA** | **p-value** |
|---|---|---|---|
| **Number of patients** | **10** | **9** | NA |
| **Median Age [range]** | 52 [30-66] | 63 [28-70] | 0.06 |
| **Sex, n (%)** | | | 0.06 |
| Male | 6 (60) | 4 (44) | |
| Female | 4 (40) | 5 (56) | |
| **Median % of predicted FEV1 [range]** | 100 [70-130] | 71 [37-109] | 0.38 |
| **Median ACQ [range]** | 0 [0-0] | 9 [4-21] | NA |
| **Inhaled Corticosteroids, n (%)** | 0 (0) | 9 (100) | NA |
| **Inhaled Salbutamol on demand, n (%)** | 0 (0) | 9 (100) | NA |
